# Documentation of delirium in the VA electronic health record

**DOI:** 10.1186/1756-0500-7-208

**Published:** 2014-04-03

**Authors:** Carol Hope, Nicollete Estrada, Charlene Weir, Chia-Chen Teng, Kavitha Damal, Brian C Sauer

**Affiliations:** 1 University of Utah and SLC Veterans Affairs Medical Center, VA Salt Lake City Health Care System, 500 Foothill Drive Bldg 182, Salt Lake City, UT 84148, USA

## Abstract

**Background:**

Delirium is a life-threatening, clinical syndrome common among the elderly and hospitalized patients. Delirium is under-recognized and misdiagnosed, complicating efforts to study the epidemiology and construct appropriate decision support to improve patient care. This study was primarily conducted to realize how providers documented confirmed cases of delirium in electronic health records as a preliminary step for using computerized methods to identify patients with delirium from electronic health records.

**Methods:**

The Mental Health Consult (MHC) team reported cases of delirium to the study team during a 6-month study period (December 1, 2009 - May 31, 2010). A chart extraction tool was developed to abstract documentation of diagnosis, signs and symptoms and known risk factors of delirium. A nurse practitioner, and a clinical pharmacist independently reviewed clinical notes during each patients hospital stay to determine if delirium and or sign and symptoms of delirium were documented.

**Results:**

The MHC team reported 25 cases of delirium. When excluding MHC team notes, delirium was documented for 5 of the 25 patients (one reported case in a physician’s note, four in discharge summaries). Delirium was ICD-9 Coded for 7 of the 25 cases. Signs and symptoms associated with delirium were characterized in 8 physician notes, 11 discharge summaries, and 14 nursing notes, accounting for 16 of the 25 cases with identified delirium.

**Conclusions:**

Documentation of delirium is highly inconsistent even with a confirmed diagnosis. Hence, efforts to use existing data to precisely estimate the prevalence of delirium or to conduct epidemiological studies based on medical records will be challenging.

## Background

Delirium is a serious neuropsychiatric condition characterized by sudden and fluctuating confusion and disorientation [1-4]. The prevalence of this neurological disorder is known to increase with age, and nearly 50% of patients over the age of 70 experience episodes of delirium during hospitalization [5-7]. Previous studies have shown associations of delirium with morbidity and mortality, as well as lengthened hospital stays [8-11]. Needless to say, delirium is a “quiet epidemic” that severely affects the quality of life of the elderly and imposes a heavy economic burden on the society [12-14]. Delirium continues to be a challenging clinical condition to treat in acute care settings, given that it is under-diagnosed in almost two thirds of cases [15-17], or it is misdiagnosed as depression or dementia [18-20]. In our aging society, this concern needs immediate attention, since delirium determines prognosis for older patients and serious adverse outcomes often trail the severity and duration of delirium during hospitalization [1,5,21,22]. In addition, since the most common causes of delirium are reversible, recognition will enhance early intervention.

Early diagnosis of delirium may lead to rapid improvement with decreased length of admission and reduced long-term complications, although the relationship between diagnosis and prognostic implications remains controversial [2,18]. Nevertheless, diagnosis is often delayed [23], and problems remain with recognition and documentation of delirium by nurses [10,24-26], and by physicians [27-29]. Although there are no definitive quantitative markers available to diagnose delirium, qualitative tools such as the Confusion Assessment Method (CAM) [30] and modified Richmond Agitation and Sedation Scale (mRASS) [31] have been validated. Unfortunately, the use of CAM and the mRASS is not ubiquitous as nurses often simply record the patient’s mental status in narrative. Understanding how delirium is recorded in the medical record is important in order to support the development of analytic strategies and tools that can provide measurement and early detection.

This study aims to determine how diagnosed cases of delirium are documented in the electronic health record. The knowledge gained from this study will support the design of a surveillance approach to monitor delirium events from electronic healthcare data. To this end, we conducted this pilot study to investigate the location, and frequency of documentation of diagnosis, and signs and symptoms of delirium, and examined the frequency of use of ICD-9 codes that represent delirium in electronic health records (EHR).

## Methods

The study used a stimulated reporting design to identify delirium cases. We then conducted chart-review on historical data (data generated during normal care process) to determine how delirium was documented in the EHR. The study period was from December 1, 2009 to May 31, 2010. The University of Utah and Salt Lake City Veterans Affairs International Review Board and Research Committee approved this study.

### Reference standard

The Delirium Mental Health Consult (DMHC) team was asked to identify patients with delirium who were referred for consultation related to cognitive functioning, adjustment to illness and management of psychiatric symptoms. The DMHC is an interdisciplinary team consisting of representatives from Psychiatry, Psychology and Clinical Pharmacy departments and uses a consult-liaison model to provide mental health services to hospitalized patients in Acute Medicine, Surgery, Neurology and Intensive Care Units. The DMHC team was asked to relay a list of patients diagnosed with delirium at bedside to the study investigators daily between December 1, 2009 and May 31, 2010. Weekly meetings between members of DMHC and the study team enabled follow-up and review of patients identified with delirium. The DMHC-referred veterans with a “bed-side” diagnosis of delirium were treated as the “reference” standard for this descriptive study.

### Chart review

Charts of the “reference” cases were thoroughly examined to distinguish the textual documentation of delirium diagnosis and its signs and symptoms.

#### Training phase

The clinical pharmacist reviewed the Unified Medical Language System (UMLS) meta-thesaurus ontologies and other published sources to develop a list of signs, symptoms and synonyms used to express delirium. The list was used to assist the two chart reviewers when reviewing clinical notes.

The clinical pharmacist, nurse practitioner and study investigators developed a chart abstraction form in excel that captured terms used to describe delirium and risk factor information found in Table 1. Five cases were reviewed as a team to discuss the abstraction process and refine the chart abstraction tool.

**Table 1:** ICD-9 codes used to represent delirium

	**Explanation**
290.3	Senile dementia with delirium
291	Delirium with alcohol withdrawal
291.1	Delirium due to alcohol (chronic)
292	Drug abuse mental disorder
292.81	Delirium induced by drug
293	Delirium due to conditions classified elsewhere
293.1	Sub-acute delirium
780.09	Delirium not otherwise specified

#### Evaluation phase

A clinical pharmacist and nurse practitioner independently reviewed the DMHC notes, physician charts, nursing notes, discharge summaries and ICD-9 billing codes of every “reference” patient. Then, patient documents were classified into having 1) a diagnosis of delirium, 2) symptoms suggestive of delirium but no stated diagnosis, or 3) no diagnosis or symptoms or signs of delirium. A diagnosis of the illness was recorded only if it was explicitly mentioned in the notes (e.g., a note that stated, “Symptoms looked like a possible case of delirium”, was classified as having symptoms without diagnosis of delirium). Chart abstraction results from the clinical pharmacist and nurse practitioner were compared for discrepancies. When discrepancies were identified the chart-reviewers (CH and NE) and study investigators (BCS) reviewed each case as a team and came to a consensus to resolve the discrepancy.

### Coded data

International Classification of Disease 9^th^ Revision (ICD-9) codes associated with the discharge summary were used as additional indicators of delirium. Codes representing delirium were not treated as a confirmed diagnosis unless a clear diagnosis was found in the medical notes. The ICD-9 codes representative of delirium were extracted from discharge diagnostic codes (Table 1). ICD-9 code indicating underlying psychiatric diagnosis that are associated with delirium and dementia such as 296 (Bipolar Disorder), and others, were excluded from analysis to reduce over-interpretation of our results.

### Statistical analysis

All descriptive statistics were conducted using SAS 9.2 software. The primary analysis presents a tabulation of the number of DMHC reported delirium cases with a documented delirium diagnosis and/or signs and symptoms of delirium in the EHR. The numbers of patients meeting each criterion were presented by document type, which included DHM consult notes, physician notes, nursing notes, discharge summaries and ICD-9 codes. Due to the exploratory nature of this study and the low sample size we did not report comparison statistics and p-values between patients with some form of documentation of delirium and patients without any documentation. Nevertheless, we present the means and frequencies of demographic and clinical variables known to be associated with delirium for the entire population to elucidate underlying patient characteristics.

## Results

Overall, 25 patients were reported by the DMHC as having delirium, of which 11 (44%) included a clear diagnosis of delirium in the DMHC note and 13 (52%) had a diagnosis or relevant signs and symptoms documented. A delirium diagnosis was documented in at least one type of EHR note in 14 (56%) and signs or symptoms documented in 16 (64%) of the 25 patients (Table 2).

**Table 2:** Documentation of delirium in patients’ charts

**Document type**	**Diagnosis**	**Signs/symptoms**	**Diagnosis or symptoms**
DMH consult notes	11 (44%)	13 (52%)	13 (52%)
Physician notes	1 (.06%)	8 (32%)	8 (32%)
Discharge summary	4 (16%)	11 (44%)	11 (44%)
Nursing Notes	-	14 (56%)	14 (56%)
ICD-9 diagnosis code	7 (28%)	-	7 (28%)
Unique veterans including DMHC notes	14 (56%)	16 (64%)	16 (64%)
Unique veterans excluding DMHC notes	9 (36%)	16 (64%)	16 (64%)

When not considering DMHC documentation, eight (32%) physician notes had documentation of a diagnosis or signs and symptoms of delirium while only one patient (4%) had a delirium diagnosis documented explicitly. Eleven (44%) discharge summaries included either a delirium diagnosis or associated signs and symptoms. While fourteen (56%) nursing notes included signs or symptoms of delirium, none included an explicit diagnosis of delirium. ICD-9 codes for delirium were coded for 7 (30.4%) of the 25 patients identified with delirium. When excluding the DMH consult notes from the analysis there was an explicit diagnosis of delirium in 9 (36%) patients and signs and symptoms in 16 (64%) of the 25 cases.

Table 3 restricts documentation to non-DMHC notes and presents the frequency of demographics variables and medical conditions between patients with documentation of delirium or signs and symptoms and those without documentation.

**Table 3:** Population demographics and medical conditions between patients with and without documented diagnosis or symptoms of delirium

	**Documented diagnosis or symptoms (n = 16)**	**No documentation (n = 9)**
**Demographics**		
Age [Mean (SD)]	68.4 (11.97)	71.0 (12.18)
Male [n (%)]	15 (93.8)	9 (100.0)
Smoker [n (%)]	8 (50.0)	3 (33.3)
Substance abuse [n (%)]	12 (75.0)	5 (55.6)
Opioids [n (%)]	3 (18.8)	1 (11.1)
Benzodiazepines [n (%)]	5 (31.3)	3 (33.3)
**Medical conditions**		
Dementia [n (%)]	2 (12.5)	3 (33.3)
CAD or ACS [n (%)]	3 (18.8)	7 (77.8)
Diabetes [n (%)]	5 (31.3)	4 (44.4)
Digestive disorders [n (%)]	9 (56.3)	7 (77.8)
Pulmonary disorders [n (%)]	6 (37.5)	6 (66.7)
Renal disorder [n (%)]	13 (81.3)	7 (77.8)
Surgery [n (%)]	2 (12.5)	3 (33.3)
Psychiatric conditions* [n (%)]	14 (87.5)	8 (88.9)
Febrile [n (%)]	3 (18.8)	2 (22.2)
Urinary tract infection [n (%)]	5 (31.3)	3 (33.3)
Other infection [n (%)]	7 (43.8)	5 (55.6)
Admitted from ECF [n (%)]	2 (12.5)	4 (44.4)
Admitted from private home [n (%)]	12 (75.0)	4 (44.4)
Discharged ECF [n (%)]	6 (37.5)	5 (55.6)
Discharged private home [n (%)]	8 (50.0)	4 (44.4)
Mechanical tube feeding [n (%)]	5 (31.3)	3 (33.3)
Patient required physical restraints [n (%)]	3 (18.8)	3(33.3)

*PTSD psychiatric conditions = schizophrenia, and/or depression, *CAD* coronary artery disease, *ACS* acute coronary syndrome, ECF extended care facility.

## Discussion

The 25 patients included in the study had delirium as determined by MHC team during bedside visits with the patient. The MHC is only conducted based on primary provider requests and not all patients evaluated by the team will have a confirmed diagnosis of delirium. However, those patients referred to the study had all been assessed as having delirium by the MHC service. The MHC notes are the status quo and the diagnosis is generally posted in the mental health consult notes or in the mental health follow-up consult notes in the VHA electronic health record (CPRS). During this study, the MHC team reported the diagnosis of delirium via email to the research team members after their consultation with the patient at his/her bedside. Despite this firsthand confirmation of delirium, medical records in nine of the 25 patients did not match their bedside diagnosis. The study team clarified this discrepancy with the MHC team and ascertained that the nine patients indeed had delirium, although this was not explicitly documented in the MHC notes. Through our pilot study, we identified that nearly one-third of the patients with bedside confirmation of delirium did not have their diagnosis reach the patient’s medical records.

Thus, the results here provide a glimpse into problems with the documentation of delirium in an electronic health record. The key findings are the lack of clear and consistent documentation across all sources, including the MHC team, physician’s notes and ICD-9 codes. The diversity and heterogeneity of the documentation process suggests a lack of shared understanding of how to represent, classify and communicate acute mental status changes.

There are several possible reasons for the source of this lack of concordance between bedside diagnosis and documentation. One is the timing involved in making an official diagnosis. Symptoms for delirium are hard to detect without formal assessment because they come and go by definition. One clinician may note a mental status change one time, but the next clinician may not. A second possible reason is the poor communication patterns in hospitals, especially between doctors and nurses. Physicians report rarely reading nurses’ notes and nurses report that if they want physicians to know something, they must communicate it verbally as it is also their experience that physician do not read their notes as well (Weir, unpublished). Finally, a third possible reason for low rates of concordance is lack of knowledge. In studies examining physicians’ and nurses’ knowledge of delirium, both roles suffer significant deficits [32].

### Implications for epidemiological studies

The lack of concordance also has implications for epidemiological studies. While clinicians have argued the prognostic implications of early diagnosis of delirium (whether it may be beneficial or not), the heterogeneity of information and clinical diagnostic process makes it impossible to arrive at conclusions. Our pilot study illustrates the dearth of documentation on delirium in medical records despite first-hand diagnosis confirmation by the primary provider (who referred the patient to the DMHC based on subjective symptoms) and the DMHC member (who ascertained the delirium diagnosis). This provides a snap-shot on how under-reported the condition is, which essentially makes it very difficult to construct any epidemiological studies to examine the disease in detail.

Ironically, although tools such as CAM exist to aid in the diagnosis of delirium, the DMHC team did not employ such tests in any of the 25 documented cases. In a prospective validation study of the CAM tool, Inouye and colleagues [33] noted that a substantial number of delirium cases go undiagnosed primarily due to missing chart documentation of symptoms. Yet, most of these tools are not robust-enough to stimulate routine use by MHC community for diagnostic purposes. It is obvious that the chain of communication regarding signs and symptoms between providers is essential not only for management of delirium in an individual patient, but also to expand our understanding of the etiology, epidemiology and to identify biomarkers/construct effective decision-support tools for delirium such that the quality of patient-care can be improved.

### Implications for informatics solutions

Even within the 25 patients examined, the variation in terminology used by each provider in the free-text narrative to document the signs and symptoms of delirium was far from standardized. Hence, mapping the ontology of the disease and using Natural Language Processing (NLP) methods to extract data for population-level observational research and analysis might not be feasible. Given the pressing demands in the health care industry to enhance quality of care in the face of cost-cutting measures, improvement of the EHR by standardizing terminologies and constructing structured questionnaires to more accurately document diagnosis, especially for chronic diseases such as delirium, will be an extremely shrewd measure to avoid the economic burden imposed by inappropriate treatments.

### Limitations

While we received DMHC reports consistently through the study period, these consultations relied upon requests made by the patient’s provider. Hence, the study was limited to cases where a DMHC consultation was ordered, leading to the small sample size. Despite the small sample size, we were able to identify the sub-standard documentation of delirium and the discrepancy between EHR and MHC notes, however, we are not able to report any significant results to substantiate our findings – instead we simply described the population studied. The “reference” patients chosen for this study were primarily confirmed with a delirium diagnosis by DMHC members, however, we are not aware of implementation of any screening tools such as CAM or NEECHAM to confirm the diagnosis, which could have further resulted in over or under estimation of patients with delirium. The study depended on stimulated reporting of identified delirium cases by DMHC team members; there is quite a possibility that some cases could have been missed, especially if such consults occurred over weekends or holidays.

## Conclusion

Our results demonstrate the inadequacy in the diagnosis and documentation of delirium in medical records, irrespective of whether the diagnosis and documentation are made by a general provider or a DMHC specialist. Any efforts to use existing data to measure prevalence, or test hypothesis on associations with other comorbidities will be difficult due to the scarcity of proper documentation as well as the inconsistencies in terminology used to represent signs/symptoms or diagnosis of delirium in the EHR.

## Competing interests

All authors declare that they have no competing interests.

## Authors’ contribution

Concept and Design: CH, BCS, CW, NE. Chart Review: CH, BCS, NE. Data Analysis: BCS, JT, CH, KD. Drafting Manuscript: KD, BCS, CW, CH. Accountable for all aspects of the work: BCS. All authors read and approved the final manuscript.
